# Hybrid Assembly of the Quorum-Quenching Isolate Variovorax paradoxus VAI-C Genome Sequence

**DOI:** 10.1128/MRA.00265-21

**Published:** 2021-05-13

**Authors:** Christopher J. Ne Ville, Jared R. Leadbetter, Paul M. Orwin

**Affiliations:** a Department of Biology, California State University San Bernardino, San Bernardino, California, USA; b Divisions of Geological & Planetary Sciences and Engineering & Applied Science, California Institute of Technology, Pasadena, California, USA; University of Southern California

## Abstract

Variovorax paradoxus VAI-C was isolated due to its ability to utilize acyl-homoserine lactones (AHLs) as the sole source of carbon, energy, and nitrogen. Here, we present a hybrid assembly of the V. paradoxus VAI-C genome sequence, consisting of a primary chromosome, a secondary chromid, and a plasmid.

## ANNOUNCEMENT

Variovorax paradoxus VAI-C was previously isolated (1) from soil adjacent to the Bowen Science Building at the University of Iowa, based on its ability to utilize the quorum-sensing signal molecule *N*-(3-oxohexanoyl)-l-homoserine lactone (3OC6-HSL; “Vibrio fischeri autoinducer-1”) as the sole source of carbon and energy. That study was also the first demonstration of quorum quenching (acyl-homoserine lactone [AHL] signal inactivation) by the acylase mechanism (1, 2). Other V. paradoxus strains also utilize AHLs (3), but not as rapidly as strain VAI-C does. Other species of bacteria also grow utilizing AHL substrates, albeit more slowly, via the same AHL acylase mechanism (4–6).

A pure culture of V. paradoxus VAI-C from a frozen stock was streaked onto a yeast extract (YE) agar plate (5 g/liter YE; Fisher Scientific). A culture derived from a single colony from this streak plate was grown at room temperature overnight in YE broth, and DNA was purified using the high-molecular-weight DNA protocol outlined for Escherichia coli (https://www.protocols.io/view/ultra-long-read-sequencing-protocol-for-rad004-mrxc57n) (7). The genomic DNA quantity and quality were assessed spectrophotometrically using the NanoDrop 1 (Thermo Fisher). Moderate shearing of the DNA was performed using a sterile 26-gauge needle (Thermo Fisher); libraries were prepared using the rapid barcoding kit (catalog number SQK-RBK004) and sequenced using an MIN-106 flow cell (R9.4.1) in an Oxford Nanopore MinION instrument. Four sequencing runs were performed on barcoded libraries derived from the same genomic sample in separate flow cells. These data were combined for assembly after demultiplexing and base calling. For all subsequent data-processing steps, default parameters were used unless otherwise noted. MinION reads were base called in Guppy v2.3.1 using the Flipflop v1.1.0 (currently referred to as Flappie or high-accuracy base calling [HAC]) model and demultiplexed in Deepbinner v0.2.0 (8). Barcodes and adapters were removed using Porechop v0.2.4 (8). A total of 194,423 Nanopore reads were obtained for V. paradoxus VAI-C (average read length, 4,557.57 ± 5,302.50; coverage, 93.88×).

The same DNA sample with additional needle shearing was used to generate a 250- to 300-bp library with the Nextera DNA Flex library preparation kit (LPK), which was sequenced on the Illumina iSeq platform (2 × 150 bp). A total of 4,818,968 Illumina reads were obtained for V. paradoxus VAI-C (average read length, 132.20 ± 30.34; coverage, 67.49×) FastQC v0.11.8 was used for quality assessment of these data (9), and trimming was performed in Trimmomatic v0.38.0 (10). Assemblies of V. paradoxus VAI-C were created using a hybrid approach in Unicycler v0.4.8.0 (11) on the North America Galaxy hub (http://usegalaxy.org) (12). The final circularization of the genomes was completed using Unicycler.

The V. paradoxus strain VAI-C genomic DNA was assembled into three circular contigs, a 6,666,455-bp primary chromosome, a 2,479,635-bp chromid, and a 292,938-bp plasmid. The Prokaryotic Genome Annotation Pipeline (PGAP) by NCBI (13) identified 8,575 protein-coding open reading frames across the three contigs, along with 61 predicted RNA genes. Replication and partition machinery (ParAB or RepAB) was identified in all three contigs, and putative conjugal transfer machinery was identified near the replication locus on the chromid. The three contigs have the following G+C contents: 69.57% for the chromosome, 68.56% for the chromid, and 60.6% for the plasmid. The average G+C content for the overall genome is 69.0%. Three incomplete prophage elements and one questionable prophage-like element were identified using PHASTER (https://phaster.ca/) (14). Eight loci were annotated as encoding penicillin acylases with homology to previously identified AHL acylase proteins from Pseudomonas aeruginosa (PA0305, HacB; PA1032, QuiP; and PA2385, PvdQ). The availability of this genome sequence will permit further investigation of the variation in quorum-quenching activity among *Variovorax* isolates.

There is substantial diversity in genome structure in the genus *Variovorax* (3, 15). The reported strain VAI-C genome assembly is the largest finished Variovorax paradoxus genome sequence and is the only assembly that contains a putative chromid and plasmid.

### Data availability.

The assemblies and sequence data have been uploaded to the NCBI database. Variovorax paradoxus VAI-C can be found under BioProject number PRJNA667957, BioSample number SAMN16392950, and assembly numbers CP063166 through CP063168. The read data can be found under SRA accession numbers SRX9260397 and SRX9260396, including demultiplexed fastQ files with the barcodes removed for the MinION runs and paired fastQ files for the Illumina iSeq.
